# Elizabethkingia anophelis Infections: A Case Series From a Tertiary Care Hospital in Uttar Pradesh

**DOI:** 10.7759/cureus.32057

**Published:** 2022-11-30

**Authors:** Nishtha Singh, Ashima Singh, Prashant Gupta, Avinash Agarwal

**Affiliations:** 1 Department of Microbiology, King George's Medical University, Lucknow, IND; 2 Department of Microbiology, Shree Guru Gobind Singh Tricentenary (SGT) University and Hospital, Gurugram, IND; 3 Department of Critical Care Medicine, King George's Medical University, Lucknow, IND

**Keywords:** e. anophelis, elizabethkingia anophelis, diagnosis, nosocomial infection, intensive care unit, empirical antibiotic therapy

## Abstract

*Elizabethkingia anophelis *is a gram-negative, aerobic, non-motile rod belonging to the ​​​​​*Flavobacteriaceae *family. *Elizabethkingia* is a genus of bacteria commonly found in the environment worldwide and has been detected in soil, river, water, and reservoirs. Over the period, it has emerged as an opportunistic human pathogen involved in neonatal meningitis and sepsis, as well as nosocomial outbreaks in adults with underlying medical conditions, including malignancies, diabetes, and chronic obstructive pulmonary disease. Here, we present a series of three cases of infection of *E. anophelis *in different clinical samples. These three cases were referred from different departments of King George's Medical University (KGMU), Lucknow, India to the Critical Care Medicine Department of KGMU, and finally succumbed to the infection.

## Introduction

*Elizabethkingia anophelis* is an aerobic, non-motile, gram-negative bacillus belonging to the *Flavobacteriaceae *family*.* The four species in this genus are *Elizabethkingia meningoseptica, E. anophelis, Elizabethkingia miricola*, and *Elizabethkingia endophytica*, with the first three being considered to be medically important [1-4]. Initially, in 2011, *E. anophelis* was isolated from the midgut of the mosquito *Anopheles gambiae* [5]. *E. anophelis* is ubiquitous and found in water, soil, and healthcare settings [6,7]. The first case reported by Frank et al. (2013) was of meningitis by *E. anophelis *in an eight-day-old girl, who was delivered by cesarean section in the Central African Republic. It was also reported to cause a nosocomial outbreak in two intensive care units from a hospital in Singapore in 2012 [8]. After the first case was reported, more cases of *E. anophelis *infection were reported from Hong Kong and Taiwan. *E. anophelis* is now recognized as the dominant *Elizabethkingia* species found in blood cultures [3]. The infections associated with *E. anophelis* are usually seen in immunocompromised patients with high mortality and rarely in healthy people [3]. Here, we present a series of three cases where *E. anophelis* was isolated from different samples suggesting it is an emerging pathogen.

## Case presentation

Case 1

**Figure 1:**
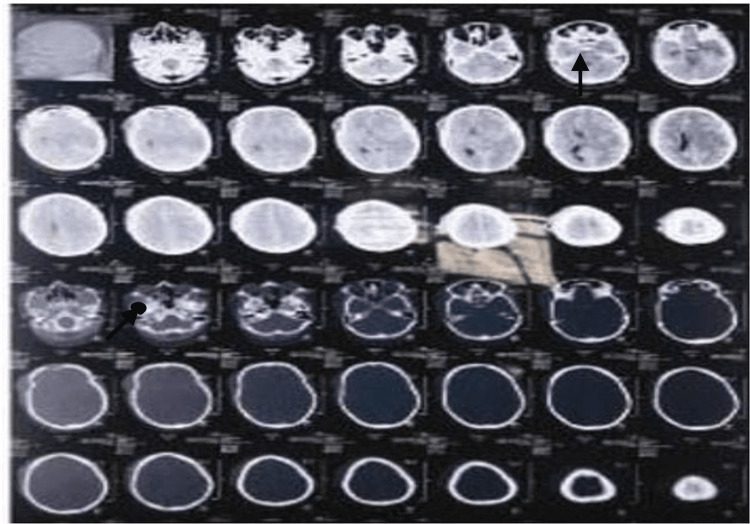
CT scan of the head showing left hemispheric acute subdural hemorrhage with a left temporoparietal contusion, shown by arrows in the figure. The first arrow shows hemorrhage and the second arrow below shows contusion

**Figure 2:**
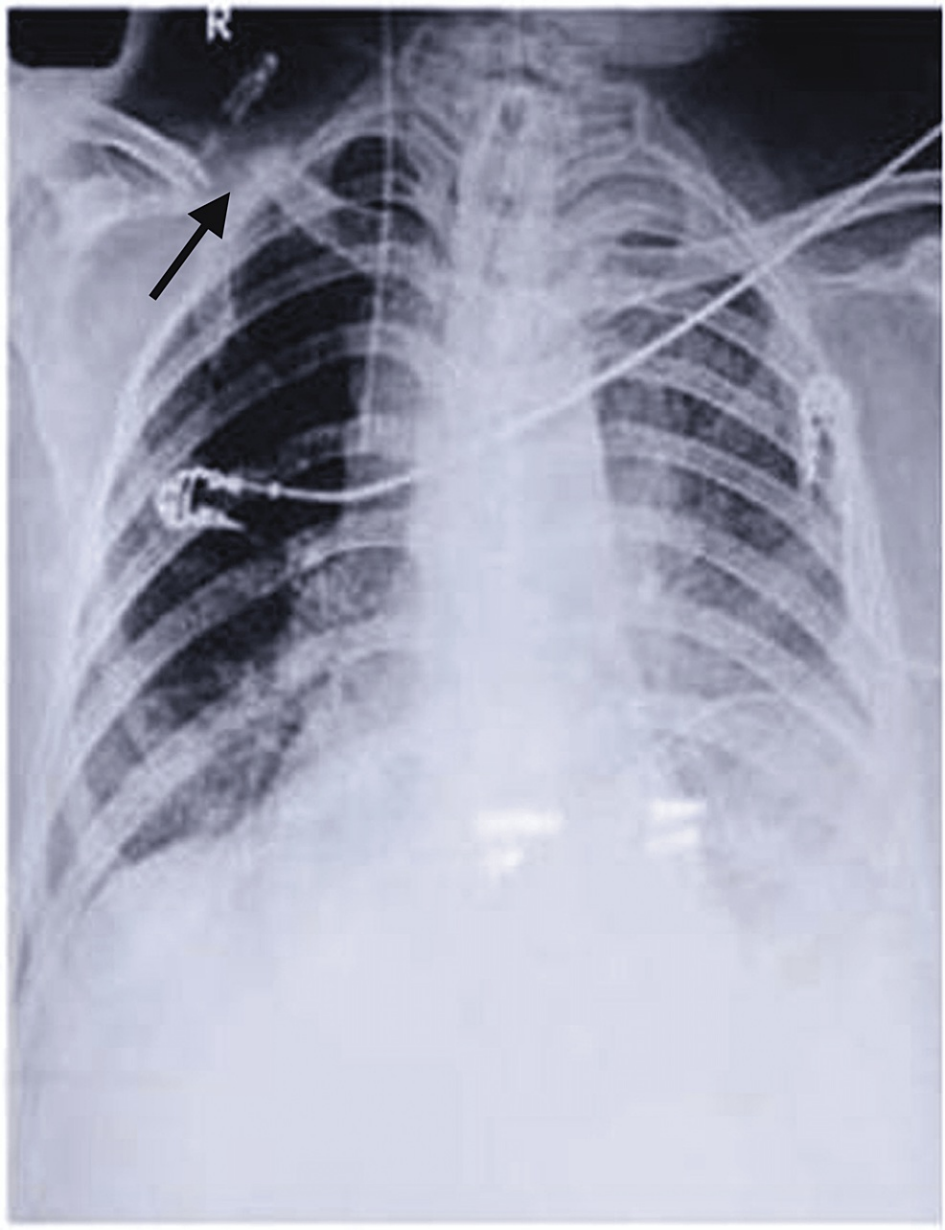
Chest X-ray showing right clavicle fracture (arrow)

A 32-year-old female, a resident of Lucknow, India, was admitted to the neurosurgery department with a complaint of a fall from riding a bike resulting in head trauma and ear bleeding. There was no history of bleeding from any other site, no seizure, or vomiting, but altered sensorium was present. Non-contrast computed tomography (NCCT) of the head showed left hemispheric acute subdural hemorrhage with left temporal-parietal contusion (Figure 1). An X-ray of the chest also revealed a right clavicular fracture (Figure 2). The patient was operated on by the neurosurgery team of doctors on the same day. Surgery performed was left frontotemporal parietal four-quadrant craniotomy with evacuation of contusion with left tempo-parietal lobectomy with augmentation duraplasty using the pericranial plate. After that, the patient was stable and was shifted to the neurosurgery ward. The patient underwent tracheostomy for elective airway management due to a decreased oxygen saturation (SpO2) of <80%, respiratory rate of 30 breaths/minute, and a fraction of inspired oxygen (FiO2) of <300 mm of Hg. The patient did not regain consciousness even after a few days of surgery and developed sepsis, and was unresponsive to the stimulus (pain or light), due to which the patient was transferred to the critical care medicine (CCM) unit for further management. In CCM, the patient was put on a mechanical ventilator along with central and peripheral lines placed. The patient was on nasogastric feed along with a tracheostomy tube. Blood culture, urine culture, and tracheal aspirate culture were sent to the microbiology department for investigation.

Investigation

Patient sample: Tracheal aspirate, urine, and a set of blood culture bottles were sent to the microbiology lab. A serum test for procalcitonin with other routine hematological investigations was done. Both the blood culture bottles flagged positive and the direct gram stain from both the blood culture bottles showed gram-negative bacilli. The culture was put on nutrient agar, blood agar, and MacConkey agar. The colonies for both plates on nutrient agar were smooth, yellow, translucent, shiny, and oxidase positive. On MacConkey agar, there was no growth. These were identified as *E. anophelis* on matrix-assisted laser desorption ionization-time of flight (MALDI-TOF) mass spectrometry. The isolate was put on Vitek MS (BioMérieux, Marcy-l'Étoile, France) for antibiotic susceptibility patterns. It was found to be susceptible to minocycline (minimum inhibitory concentration (MIC) ≤ 1) and trimethoprim-sulfamethoxazole (MIC ≤ 2/38). Procalcitonin was 17.67 ng/ml and hematological investigation showed an increase in total leukocytes count. The urine culture was sterile and the tracheal aspirate culture showed heavy growth of *Acinetobacter baumannii, *colony count > 10^5 ^CFU/ml, which was found to be susceptible to meropenem, tetracycline, and colistin. Colistin (MIC = 0.75 ug/ml) was done by the broth microdilution method.

Treatment

The patient was on colistin 2 m.i.u IV injection three times a day (TDS), meropenem 500 mg injection twice a day (BD), trimethoprim 500 mg IV injection BD, and Lasix ½ ampule injection BD, and other supportive treatment was given.

Outcome and Follow-Up

The patient succumbed to illness after being treated for about one month and developed respiratory distress and cardiac arrest leading to death.

Case 2

A 60-year-old female, a resident of Lucknow, was admitted to the Emergency Department of Cardiology, King George's Medical University (KGMU) with complaints of deviation of angle of the mouth, history of altered sensorium, right-side hemiparesis, slurring of speech, and breathlessness for 12 days. An echocardiogram (ECHO) performed on the same day showed concentric left ventricular hypertrophy for which emergency treatment was given. CT scan of the head performed two days after revealed a right-sided cerebral infarct for which surgery was planned, but due to severe breathlessness and respiratory distress (SpO2 < 85%, FiO2 > by more than 0.2%, and respiratory rate > 30 breaths/minute), she could not be operated. She was shifted to the CCM department for an emergency tracheostomy along with central lines intact. Blood culture and tracheal aspirate were sent to the microbiology department for investigation, as fever and other signs of sepsis were present.

Investigation

**Figure 3:**
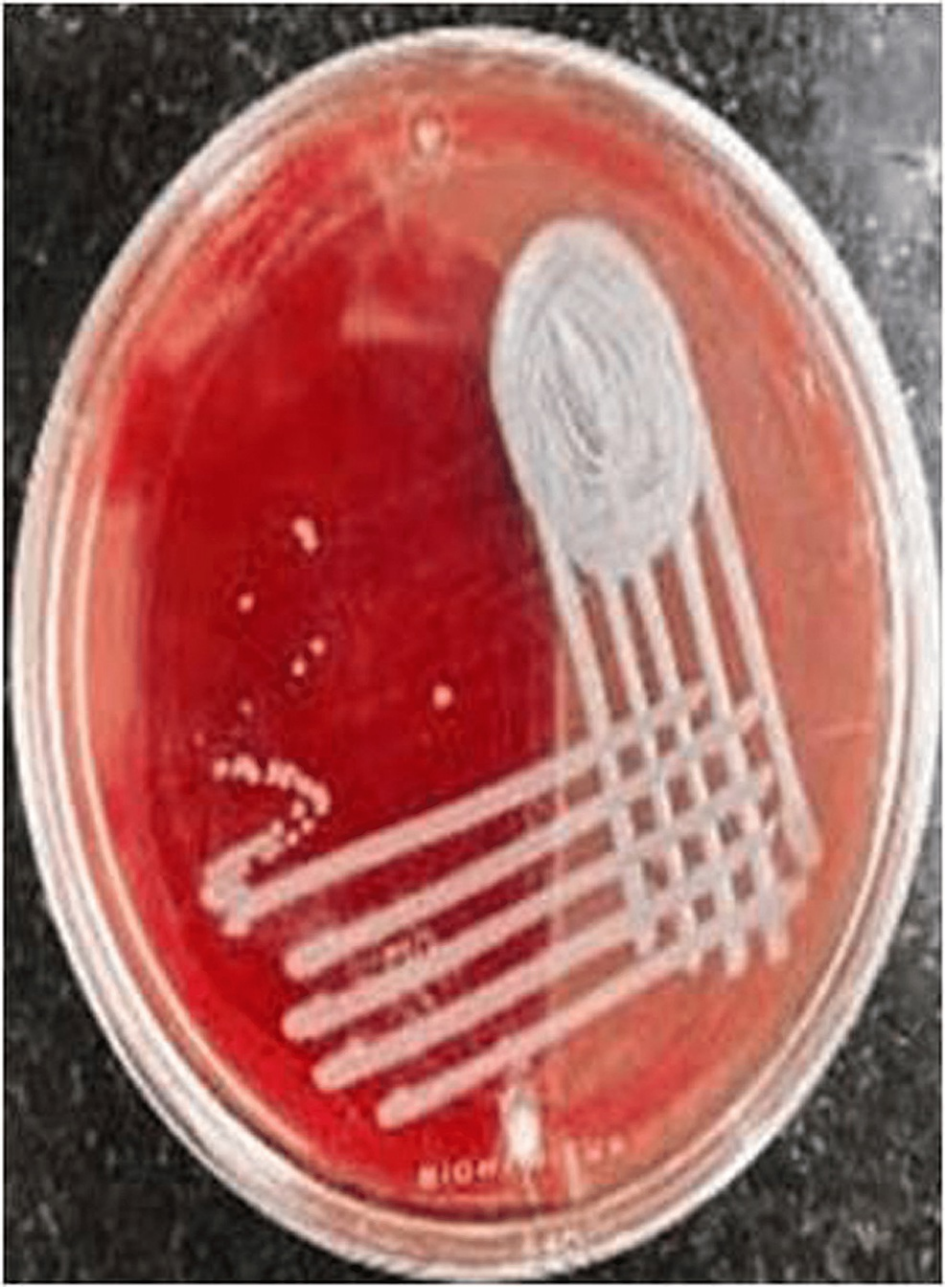
Colonies of Elizabethkingia anophelis on blood agar

Patient sample: Tracheal aspirate and a set of blood culture bottles were sent to the microbiology lab for evaluation and a blood sample for routine hematological investigations was also sent. Both of the blood culture bottles flagged positive and the direct gram stain from the bottle showed gram-negative bacilli. Subculture from the bottle was put on the blood agar, nutrient agar, and MacConkey agar. After 24 hours of aerobic incubation at 37°C, the colonies on the blood agar were light yellow, translucent, and shiny in nature (Figure 3). There was no growth on MacConkey agar. These yellow translucent colonies were oxidase and catalase positive, which were identified as *E. anophelis* by MALDI-TOF mass spectrometry (Vitek MS). The antibiotic susceptibility performed showed susceptibility only to cotrimoxazole. Her tracheal culture showed the growth of *Klebsiella pneumoniae*, which was found to be multi-drug resistant, and susceptible to meropenem and colistin. Investigations of the patient are listed in Table 1.

**Table 1:** Investigations

Test name	Result	Units	Normal range
Hemoglobin	8.653	gm/dl	(12-16)
Total leucocytes count	19,820	cells/cumm	(4,000-11,000)
Neutrophils	85	%	(40-70)
Lymphocytes	12	%	(20-40)
Eosinophils	01	%	(01-06)
Monocyte	02	%	(0-02)
Basophils	00	%	(0-01)
Platelet count	1.5	Lakh cells/cumm	(1.5-4.5)
Total RBC count	3.045	Million cells/ul	(3.5-5.5)
MPV (mean platelet volume)	12.43	fl (femtoliters)	(7.0-10.4)
MCV (mean cell volume)	92.2	fl (femtoliters)	(76-96)
MCH (mean corpuscular hemoglobin)	28.42	pg	(27-32)
MCHC (mean corpus hemoglobin concentration)	30.82	%	(31-35)
RDW (red cell distribution width)	15.82	%	(11.5-14.5)
HCT (hematocrit)	28.07	%	(36-46)
PCT (procalcitonin)	28.6	ng/ml	<0.5 ng/ml
Serum urea	72.0	mg/dl	(12.9-42.9)
Serum creatinine	1.85	mg/dl	(0.6-1.4)
Serum sodium	142.7	mmol/l	(135-145)
Serum potassium	3.32	mmol/l	(3.5-5.3)
Serum ionic calcium	4.76	mg/dl	(4.5-5.5)

Treatment

The patient was empirically put on injection of meropenem 1 gm IV TDS and injection of clindamycin 600 mg IV TDS. After the reports of positive blood culture, the patient was started on cotrimoxazole 500 mg IV injection TDS. Injection of Lasix ½ ampule BD, injection of caspofungin 40 mg IV BD, and other supportive management were given.

Outcome and Follow-Up

Despite all of the above management, the patient suffered cardiac arrest and succumbed to the infection approximately one month of infection.

Case 3

A 65-year-old patient, a resident of Chowk, Lucknow, was admitted to the CCM, KGMU, Lucknow with chief complaints of vomiting after eating anything, decrease urine output, decrease appetite, and breathlessness in the past two months. The patient was earlier admitted to Sahara Hospital where intubation was done, as SpO2 was decreased below 80% and the patient had severe bradycardia. The patient was discharged after vitals were stable but again came to KGMU due to the above complaints. The ECHO performed on the same day showed concentric right ventricular hypertrophy for which treatment was started. The patient's breathlessness got worsened along with stridor and was reintubated again in the CCM department. Blood culture and tracheal aspirate were sent to the microbiology department for investigation.

Investigation

Patient sample: Tracheal aspirate and a set of blood culture bottles were sent to the microbiology lab for evaluation and a blood sample for routine hematological investigations was also sent. The hematological finding showed hemoglobin at 12.2 gm, total leucocyte counts at 14,000/cumm, and serum creatinine at 0.99 mg/dl. ECG showed T-wave inversion and ECHO ejection fraction of 50%. General examination revealed a pulse rate of 84/minute, blood pressure of 110/80 mm of Hg, the chest was bilaterally clear, respiratory rate was 28/minute, and SpO2 was found to be 80%. Systemic examination was found to be within normal limits. The patient was diagnosed with coronary artery disease, non-ST elevation myocardial infarction (NSTEMI), and was troponin I positive, which showed right coronary artery disease.

Microbiological investigations: The blood culture was found to be sterile after five days of incubation. The gram stain of tracheal aspirate showed plenty of pus cells along with gram-negative bacilli. After 24 hours of incubation at 37°C, the tracheal aspirate culture showed the growth of significant colonies on blood agar. The colony was light yellow, translucent, and shiny. There was no growth on MacConkey agar. These yellow translucent colonies were oxidase and catalase positive, which were identified as *E. anophelis* by MALDI-TOF mass spectrometry (Vitek MS). The antibiotic susceptibility performed by Vitek MS showed susceptibility to minocycline (MIC ≤ 1), colistin (MIC 1 ug/ml), and cotrimoxazole (MIC ≤ 1).

Treatment

The treatment empirically started with an injection of meropenem 500 mg BD IV, an injection of teicoplanin 400 mg IV BD, and an injection of caspofungin 40 mg IV BD. After the tracheal culture report, an injection of minocycline 400 mg IV once a day (OD) was given. Injection of Lasix with other supportive management was provided.

Outcome and Follow-Up

The patient did not respond well to the therapy and died due to respiratory collapse and right-sided heart failure.

## Discussion

*E. anophelis* bacteremia should be considered clinically significant unless proven otherwise, and immediate appropriate antibiotic therapy should be started as it is a multi-drug resistant organism. *E. anophelis* is an opportunistic pathogen and may cause high mortality [3]. Infections due to *E. anophelis* are not known because they are usually misidentified as *E. meningoseptica* by conventional identification methods and automated identification systems [9]. Before the upgradation of the MALDI-TOF mass spectrometry, even matrix-assisted laser desorption/ionization (MALDI) was used to misidentify *E. anophelis* as *E. meningoseptica* or *Elizabethkingia* spp. Now after the improved and upgraded database of MALDI, correct identification of *E. anophelis* is possible [10]. Even though both the species of genus *Elizabethkingia* cause diseases with similar clinical manifestations, the need to differentiate between both species of *Elizabethkingia* is advocated in the genus of *Elizabethkingia*, in view of the fact that *E. anophelis* is a source of a larger part of human infections as compared to *E. meningoseptica*. Over and above that, there are studies documenting a variation in the antimicrobial susceptibility results, also between both species of *E. meningoseptica* and *E. anophelis*. It acquires a divergent alteration in the target gene for fluoroquinolones, and there is a mutation in the amino acid in the quinolone-resistance determining regions (QRDRs) [11]. Despite investigations by the CDC in the Midwestern USA outbreaks, the routes of transmission of *E. anophelis* are still not very clear, and no evident sources or routes of disease transmission are identified [12]. Studies have shown that most patients with *Elizabethkingia* infections have chronic underlying illnesses, such as diabetes mellitus, cardiovascular disease, chronic renal disease, malignancy, and liver cirrhosis [13]. But in our study, the patients were young and immunocompetent, who had the infection due to prolonged hospital stay and succumbed to it (Table 2).

**Table 2:** Summary of case findings from our case series at the time of sample collection

	Case 1	Case 2	Case 3
Age (years)	32	60	65
Gender	Female	Female	Female
Past medical history	Subdural hemorrhage with a left temporal-parietal contusion	Left ventricular hypertrophy and right-sided cerebral infarct	Concentric right ventricular hypertrophy
Blood pressure (mmHg)	90/70	100/70	90/69
Heart rate (bpm)	120	110	60
Isolated microorganism	*Elizabethkingia anophelis* and *Acinetobacter baumannii*	*Elizabethkingia anophelis* and *Klebsiella pneumoniae*	Elizabethkingia anophelis
Pharmacological treatment	Colistin, meropenem	Meropenem, clindamycin	Meropenem, teicoplanin, caspofungin

The case fatality rate of patients with *E. anophelis* infection has been approximately 24-34% in previous reports [13]. Therefore, how to choose the correct empirical antibiotics for patients with *Elizabethkingia* infections is of immense importance [14]. Very less information is available on the antimicrobial susceptibility patterns of *Elizabethkingia*. In our case, the finding of this isolate was accidental as a rare pathogen to be isolated. The reason could be due to prolonged hospital stay and mixed microbial infection. Several studies have shown that *E. anophelis* is usually resistant to multiple antibiotics, including most β-lactams, β-lactam/lactamase inhibitors, carbapenems, and aminoglycoside, but is variably susceptible to piperacillin, piperacillin-tazobactam, minocycline, tigecycline, fluoroquinolones, and trimethoprim-sulfamethoxazole [14]. Therefore, combination therapy is recommended over monotherapy in multidrug-resistant *E. meningoseptica* and *E. anophelis* bacteremia. It has been suggested that prolonged therapy with a combination of rifampin with vancomycin, trimethoprim-sulfamethoxazole, minocycline, or fluoroquinolones may have better clinical outcomes [15]. The above findings implicate that strict infection control measures are to be followed rigorously in ICUs and other high-risk areas. Bundle care implementation and audit to be performed in patients on devices. In addition, hospitals should have a properly defined antibiotic stewardship program [16]. After analyzing various studies, we noted that inadequate empirical antimicrobial therapy can be an independent risk factor for mortality, as shown in Table 3 [9,17-20].

**Table 3:** Comparative table of various studies of isolation of Elizabethkingia anophelis from different samples and their susceptibility pattern

S. No.	Specimen	Isolate	Sensitivity pattern	References
1.	Sputum culture	E. anophelis	Susceptible to cotrimoxazole and moxifloxacin	Hu et al. (2017) [9]
2.	Tracheal aspiration	E. anophelis	Susceptible to piperacillin-tazobactam and tigecycline only	Bai and Liu (2020) [17]
3.	Blood culture	E. anophelis	Susceptible only to piperacillin-tazobactam	Mantoo et al. (2021) [18]
4.	Blood culture	E. anophelis	Susceptible to levofloxacin, trimethoprim-sulfamethoxazole, and tetracycline	Honavar et al. (2021) [19]
5.	Peritoneal fluid	E. anophelis	Susceptible to fluoroquinolones and trimethoprim-sulfamethoxazole	Kalchev et al. (2022) [20]

## Conclusions

*E. anophelis* is an emerging opportunistic pathogen in developing countries like India. Multiple risk factors, such as indwelling catheters, venous line insertion, irrational and prolonged use of broad-spectrum antibiotics, and pre-existing co-morbidities, have led to increasing numbers of cases. It is usually misidentified and it often leads to a high mortality rate because of its multiple antibiotic resistances. Therefore, *E. anophelis* infections should be considered clinically significant, and correct identification can be critical to ensure patients receive appropriate treatment.
